# Genome Sequence of a *Xylella fastidiosa* Strain Causing Sycamore Leaf Scorch Disease in Virginia

**DOI:** 10.1128/genomeA.00773-14

**Published:** 2014-08-21

**Authors:** Wei Guan, Jonathan Shao, Robert E. Davis, Tingchang Zhao, Qi Huang

**Affiliations:** a Floral and Nursery Plants Research Unit, U.S. Department of Agriculture, Agricultural Research Service, Beltsville, Maryland, USA; b Molecular Plant Pathology Laboratory, U.S. Department of Agriculture, Agricultural Research Service, Beltsville, Maryland, USA; c Institute of Plant Protection, Chinese Academy of Agricultural Sciences, Beijing, China

## Abstract

*Xylella fastidiosa* causes bacterial leaf scorch in landscape trees including sycamore. We determined the draft genome of *X. fastidiosa* strain Sy-Va, isolated in Virginia from a sycamore tree displaying leaf scorch symptoms. The Sy-VA genome contains 2,477,829 bp, and has a G+C content of 51.64 mol%.

## GENOME ANNOUNCEMENT

*Xylella fastidiosa* is a Gram-negative, nutritionally fastidious, insect-transmitted and xylem-inhabiting bacterium that causes a wide range of plant diseases, including Pierce’s disease of grapevine and bacterial leaf scorch in landscape trees such as sycamore. At present, five complete genomes of *X. fastidiosa* have been reported, including those of the citrus variegated chlorosis strain 9a5c (1), Pierce’s disease strains Temecula 1 (2) and GB514 (3), and almond leaf scorch strains M12 and M23 (4). Draft genomes have also been published for the oleander strain Ann1, the almond strain Dixon (5), the elderberry strain EB92-1 (6), and very recently for the mulberry strain Mul-MD (7) and the oak strain griffin-1 (8) of *X. fastidiosa*. We announce here determination of the draft genome of the sycamore strain of *X. fastidiosa*, Sy-VA, which was isolated in October of 2002 from a sycamore tree displaying leaf scorch symptoms in Virginia.

Genomic DNA was extracted, from a pure culture of *X. fastidiosa* strain Sy-VA grown in periwinkle wilt medium (9), using the Blood and Tissue kit (Qiagen, Inc., Valencia, CA) according to the manufacturer’s instructions. Random shotgun and 3-kb mate-pair libraries of Sy-VA were generated and sequenced using Roche 454 GS (FLX titanium) pyrosequencing, resulting in 360,445 shotgun reads and 727,509 mate-pair and single reads totaling 351,334,881 bases having a read length average of ca. 300 bases. After processing by the Newbler gsAssembler v2.7, the total number of reads from all libraries was 1,073,162 aligned reads, with 349,185,278 bases aligned. The genome was assembled into 139 contigs and 25 scaffolds using the Newbler Assembler. The average length of all the contigs was 17,826 bases. The largest contig was 342,206 bases. Among the large contigs that are greater than 600 bases, the *N*_50_ contig size was 119,240 bases. All the contigs were run through an annotation pipeline using GeneMark.hmm to predict coding regions based on prior *Xylella fastidiosa* gene models. To determine endpoints of putative protein coding genes >149 bases, BLASTx was carried out using the contigs as queries against the non-redundant protein sequences (nr). Selected open reading frames that were not consistent with annotated *Xylella* genes were manually annotated. Predictions of regions encoding tRNAs and rRNAs were made using tRNAscan-SE and RNAmmer programs, respectively.

The depth of sequencing read coverage per base position was 141× for this draft genome of the *X. fastidiosa* strain Sy-VA; the genome contains 2,477,829 bp and has a G+C content of 51.64 mol%. A total of 2,231 protein-encoding regions or putative genes were predicted, 2,168 of which were tentatively assigned a function. In addition, a ~26-kb plasmid sequence was found that is most similar to the plasmid of the grapevine-infecting GB514 strain (3) of *X. fastidiosa*.

### Nucleotide sequence accession numbers.

This whole-genome shotgun sequence has been deposited at DDBJ/EMBL/GenBank under the accession no. JMHP00000000. The version described in this paper is version JMHP01000000.
